# Strengthening Regional Control of Transboundary Animal Diseases in the Greater Mekong Subregion of Southeast Asia: Four Decades of Diagnostics, Surveillance, Field Epidemiology, and Policy Translation

**DOI:** 10.1155/tbed/5796839

**Published:** 2026-08-11

**Authors:** Stuart D. Blacksell, Peter A. Windsor, James R. Young, Syseng Khounsy, Phouvong Phommachanh, Russell D. Bush, Harish Tiwari, Jarunee Siengsanan-Lamont, Nina Matsumoto, Sothyra Tum, James V. Conlan, Somjai Kamolsiripichaiporn, Jaison B. Kolenchery, Laurence J. Gleeson, Michael P. Ward

**Affiliations:** ^1^ Mahidol Oxford Tropical Medicine Research Unit (MORU), Faculty of Tropical Medicine, Mahidol University, Bangkok, Thailand, mahidol.ac.th; ^2^ Centre for Tropical Medicine and Global Health, Nuffield Department of Medicine, University of Oxford, Oxford, UK, ox.ac.uk; ^3^ Sydney School of Veterinary Science, Faculty of Science, The University of Sydney, Camperdown, NSW, Australia, sydney.edu.au; ^4^ National Animal Health Laboratory, Department of Livestock and Fisheries, Ministry of Agriculture and Environment, Vientiane, Laos; ^5^ National Animal Health and Production Research Institute, General Directorate of Animal and Production, Department of Animal Health and Veterinary Public Health, Phnom Penh, Cambodia; ^6^ Food Standards Australia New Zealand, Canberra, Australian Capital Territory, Australia

**Keywords:** African swine fever, classical swine fever, diagnostics, foot-and-mouth disease, One Health, smallholder livestock, Southeast Asia, surveillance, transboundary animal diseases

## Abstract

Transboundary animal and emerging infectious diseases (TADs, EIDs) threaten livestock health and production, food security, and the livelihoods of smallholder farmers in Southeast Asia (SEA). Outbreaks of TADs, including foot‐and‐mouth disease (FMD), classical swine fever (CSF), African swine fever (ASF), and lumpy skin disease (LSD), highlight ongoing risks associated with informal animal movement and cross‐border trade. We reviewed four decades of research from Thailand, Lao PDR, and Cambodia in the Greater Mekong Subregion (GMS) to track the evolution of diagnostics, surveillance, and policies for these diseases. Incremental investments in laboratory diagnostics, epidemiology, abattoir surveillance, and field health measures were integrated into disease control strategies by adaptation to resource‐limited, endemic settings and shaped by regional collaboration, notably through the SEA, China and Mongolia FMD (SEACFMD) campaign. Key achievements include establishing disease prevalence baselines, improving understanding of virus dynamics, identifying local disease risk factors, identifying veterinary service gaps, and rapidly increasing diagnostic and response capacity during outbreaks. Evidence from the studies reviewed suggests that integrating diagnostics with field surveillance, socioeconomic information, and regional coordination can provide actionable insights for disease control, although implementation and effectiveness have varied between diseases, countries, and time periods. This narrative review suggests that sustainable control requires long‐term adaptive development of diagnostics, surveillance, and policy translation while recognising persistent heterogeneity in veterinary capacity, surveillance coverage, and biosecurity across the region. This review is based on a narrative synthesis of published literature, institutional reports, and programme experience.

## 1. Introduction

Major transboundary animal diseases (TADs), including foot‐and‐mouth disease (FMD), classical swine fever (CSF), and, more recently, African swine fever (ASF) and lumpy skin disease (LSD), have impacted livestock development, rural livelihoods, and transboundary disease health preparedness across Southeast Asia (SEA). With porous borders, dense smallholder systems, and an increasingly dynamic, often unregulated livestock trade in the region, ideal conditions for pathogen spread have emerged, creating a practical testing ground for disease‐control innovation.

This review summarises four decades of work spanning Thailand, Lao PDR, and Cambodia, tracing the progression from laboratory diagnostics to field epidemiology and ultimately to policy engagement and surveillance system design. The focus has been on strengthening veterinary infrastructure, developing diagnostics suited to low‐resource settings, and generating actionable epidemiological evidence to support national and regional decision‐making. Some aspects of this work have been described previously in reviews of Australian‐supported animal health programmes in SEA, including evaluations of the SEA, China and Mongolia FMD (SEACFMD) campaign [1–5].

Of particular importance to livestock disease control in the Greater Mekong Subregion (GMS) is the predominance of smallholder livestock production systems, which often operate under resource constraints and have highly variable access to veterinary services. As urban‐based economies have flourished and food preferences have altered [4], a prolonged surge in demand for animal‐sourced foods and only modest increases in local production have led to increased livestock and product movement into and out of the GMS. This was accompanied by surging risks of TAD incursions, threats of zoonoses, and antimicrobial resistance [4]. Recent epidemics in the GMS have resulted from incursions of highly pathogenic avian influenza (HPAI), new strains of FMD virus, ASF, LSD, and porcine reproductive and respiratory syndrome (PRRS). These incidents reflect inadequate biosecurity and unregulated trading in both livestock‐ and wildlife‐sourced foods, as well as variable veterinary public health capacity and regulatory implementation [4, 5].

This review synthesises four decades of research and programme experience primarily undertaken by the authors and long‐term collaborators in Thailand, Lao PDR, and Cambodia. Although the review focuses on these activities, they occurred alongside major regional contributions from Food and Agricultural Organisation (FAO), World Organisation for Animal Health (WOAH), Australian Centre for International Agricultural Research (ACIAR), Defense Threat Reduction Agency (DTRA), the European Union, China, Japan, the Republic of Korea, and national veterinary services across SEA. A comprehensive review of all regional initiatives is beyond the scope of this paper. Rather, the objective is to critically examine how diagnostics, surveillance, epidemiology, and policy translation evolved within this body of work while acknowledging remaining limitations, uncertainties, and challenges for sustainable regional disease control. To add to the recent reviews [1–5], this paper provides a reflective synthesis of field‐based research, diagnostic development, and policy translation conducted over four decades across mainland SEA. Collectively, these studies suggest that sustainable control of TADs will not result from single technical interventions but from the progressive integration of diagnostics, field epidemiology, surveillance platforms, and policy engagement within national veterinary systems.

## 2. Methods

This article presents a narrative synthesis of major TAD research conducted in SEA, including Thailand, Lao PDR (Laos), and Cambodia between 1988 and 2025. The review integrates the published literature with findings from long‐term surveillance programmes conducted by the authors and collaborating institutions in SEA. Relevant studies were identified through targeted searches of PubMed and Web of Science, supplemented by institutional project outputs and the authors’ expert knowledge of long‐running surveillance programmes in the region. Because several authors participated directly in many of the studies reviewed, interpretation may be influenced by experiential perspectives. To minimise this possibility, emphasis was placed on peer‐reviewed publications, where available, and on official reports and documented programme outputs. Contradictory findings and recognised programme limitations are discussed where appropriate; however, the review should not be interpreted as a systematic assessment of all TAD activities undertaken within the GMS.

Publications were selected based on their relevance to diagnostics, epidemiology, surveillance, laboratory development, and policy translation related to FMD, CSF, ASF, and other major TADs in Thailand, Lao PDR, and Cambodia. Preference was given to peer‐reviewed publications. Where important programme developments were not represented in the peer‐reviewed literature, institutional reports and documented project outputs were included. No formal quality assessment or meta‐analysis was undertaken.

## 3. FMD

There is an increasing body of published literature on FMD control in the GMS that addresses most aspects of the disease [5]. This includes recent papers on the history of FMD [3–5], virology and immunology [3, 6], epidemiology [7], socioeconomic impacts [8–10], vaccination strategies [3, 11–14], biosecurity [15], and strategic disease control [1, 3]. The information from these and other studies continues to inform strategic disease control initiatives. A summary of studies is presented in Table 1.

**Table 1:** Key transboundary animal disease studies conducted in Thailand, Lao PDR and Cambodia over four decades, illustrating progressive development of diagnostics, epidemiology and molecular surveillance.

Pathogen	Country	Study focus	Approach	Year(s)	Key contributions	Principal limitation	Reference(s)
Foot‐and‐mouth disease	Thailand	Antigenic characterisation and serotyping	ELISA development and antigenic comparison	1992–1995	Identified antigenic variation relevant to diagnostic selection and vaccine strain matching	Did not assess vaccine potency, delivery, or population‐level protection	[16–20]
	Thailand	Vaccine field performance	Trivalent vaccine serological evaluation	1994	Demonstrated improved serological responses following introduction of the trivalent vaccine	Did not establish long‐term field effectiveness across production systems	[21]
	Thailand	Village‐level epidemiology	Risk factor and vaccination surveys	1995–1996	Identified livestock movement, management practices, and vaccination gaps as drivers of endemic transmission	Findings were limited to the surveyed locations and production systems	[22, 23]
	Lao PDR	Outbreak impact in smallholder systems	Field investigation and economic assessment	2002	Quantified the production and economic effects of FMD in smallholder systems	Findings from the investigated outbreaks may not represent impacts nationally	[24]
	Lao PDR	National seroprevalence	Structured serosurveillance	2008	Established a national‐scale baseline of FMD exposure	Missing vaccination histories limited interpretation of serological results	[25]
	Lao PDR	Outbreak review	National control programme assessment	2008	Identified operational strengths and gaps in the national FMD control programme	Conclusions depended on the completeness and quality of available outbreak records	[26]
	Lao PDR	Molecular epidemiology	VP1 sequencing and topotype identification	2009	Identified circulating topotypes and provided evidence of transboundary virus movement	Available outbreak samples may not represent all circulating viral diversity	[27]
	Lao PDR	Spatial epidemiology	Hotspot and movement risk analysis	2013	Identified geographic hotspots and livestock‐movement risks relevant to targeted control	Risk estimates depended on the completeness of surveillance and movement data	[28]
	Lao PDR	Vaccination impact	Risk‐based vaccination trial	2010; 2022	Provided evidence supporting risk‐based vaccination in high‐risk communities	Broader effectiveness depended on coverage, vaccine quality, and sustained programme delivery	[3, 11–14]
	Cambodia	Socioeconomic impact	Financial impact assessment	2013	Quantified the substantial household economic losses associated with FMD	Estimates were based on selected affected communities and may not be nationally generalisable	[29, 29]
	Cambodia	Economic evaluation	Benefit–cost analysis of vaccination	2016	Demonstrated the potential economic value of vaccination in high‐risk areas	Results depended on assumptions about outbreak risk, vaccine performance, and programme costs	[10]
	Cambodia	Biosecurity	Farmer behaviour and biosecurity uptake	2015; 2017	Demonstrated that extension activities could improve farmer biosecurity and vaccination practices	Improvements required sustained veterinary support and were variable between communities	[15, 30]
	Lao PDR	Integrated surveillance	Abattoir‐based multidisease serosurveillance	2021–2022	Demonstrated the feasibility of cost‐effective, abattoir‐based multidisease surveillance	Limited traceability and vaccination data restricted epidemiological interpretation	[31, 32]
	Cambodia	Abattoir surveillance	NSP seropositivity mapping	2022; 2025	Generated geographic information on FMD exposure to support risk mapping	Abattoir sampling was not fully representative and could not identify active transmission alone	[33, 34]
Classical swine fever	Lao PDR	National outbreak confirmation	Analysis of clinical submissions	2001	Confirmed widespread CSF circulation and established an initial national disease baseline	Clinical submissions were influenced by passive‐reporting and sampling biases	[35]
	Lao PDR	Population seroprevalence	Structured serological surveys	2000–2001	Identified geographic variation in exposure and vaccination coverage	Incomplete vaccination records prevented clear separation of vaccination from natural infection	[35, 36]
	Lao PDR	Genetic typing	E2 gene phylogenetic analysis	2004	Identified circulating CSFV lineages and their relationships with regional viruses	The available sequences represented only a subset of circulating viruses	[37]
	Lao PDR	Genetic typing	5′ NCR molecular characterisation	2005	Confirmed the circulation of multiple CSFV genetic lineages	Analysis of a limited genomic region provided restricted phylogenetic resolution	[38]
	Lao PDR	Diagnostic optimisation	RT‐PCR and ELISA validation under field conditions	2004; 2007	Adapted RT‐PCR and ELISA methods for use in resource‐limited laboratories	Performance under study conditions may not reflect routine use across all laboratories	[39, 40]
	Lao PDR	Rapid antigen detection	Immunomagnetic bead assay evaluation	2009	Demonstrated the feasibility of simplified rapid antigen detection	Diagnostic performance required confirmation in larger and more diverse sample sets	[41]
	Lao PDR	Longitudinal surveillance	Abattoir‐based seroprevalence comparison	2023	Enabled comparison of CSF seroprevalence over approximately two decades	Missing vaccination records prevented firm conclusions about the causes of changing seroprevalence	[42]
	Thailand	Genetic typing	Emergence of genogroup 2 strains	1996; 2000	Documented the emergence and evolution of CSFV genogroup 2 strains	Available samples provided incomplete temporal and geographic coverage	[43, 44]
	Vietnam	Genetic typing	E2 and Erns molecular characterisation	2021	Documented continued evolution of CSFV lineages in the regional context	Findings from Vietnam could not be directly generalised to the wider GMS	[45]
	China	Genetic typing	Genotype 2 predominance studies	2014; 2019; 2021	Provided regional context for the emergence and predominance of CSFV genotype 2	Differences in surveillance and production systems limited direct comparison with GMS countries	[46–48]
African swine fever	Lao PDR	Rapid field diagnostics	Evaluation of antigen rapid diagnostic test	2020	Demonstrated the potential value of rapid antigen testing for field triage	Limited sensitivity meant that negative results required molecular confirmation	[49]
	Lao PDR	Livelihood impact	Smallholder socioeconomic assessment	2021	Documented the severe effects of ASF on smallholder livelihoods and household resilience	Findings from affected villages may not represent impacts across all production systems	[50]
	Lao PDR	Transmission modelling	Spatiotemporal outbreak modelling	2024	Estimated transmission patterns relevant to preparedness and outbreak response	Estimates depended on the completeness and accuracy of reported outbreak data	[51]
	Lao PDR	Policy translation	Scenario‐based outbreak modelling	2025	Demonstrated how scenario‐based modelling could support preparedness and resource allocation	Model outputs depended on assumptions and incomplete surveillance data	[52]
	Lao PDR	Postepidemic monitoring	Abattoir‐based surveillance	2023	Demonstrated the value of abattoir surveillance for monitoring postepidemic exposure patterns	Under‐reporting, selection bias, and uncertain animal origins limited interpretation	[42]

Abbreviations: CSFV, classical swine fever; ELISA, enzyme‐linked immunosorbent assay; FMD, foot‐and‐mouth disease; NCR, noncoding region; NSP, nonstructural protein; RT‐PCR, reverse transcription polymerase chain reaction.

Reflections on these studies lead to the conclusion that more effective FMD control requires the development of improved regional biosecurity and reduced ‘informal’ movement of livestock and their products throughout the GMS [5]. This widespread failure in biosecurity is accompanied by a critical issue for current attempts to increase FMD vaccination, requiring a focus on vaccine serotypes and matching, strategies and efficacy, and delivery logistics and capacities [5]. However, despite attempts to integrate activities through the SEACFMD campaign, progress in each country within the programme has varied, largely due to differences in animal health surveillance and response capacity.

A framework for the progressive control of TADs (GF‐TADs) was launched in 2004 as a joint initiative of the FAO of the United Nations and the Office International des Epizooties (OIE, now the WOAH), with the participation of the World Health Organisation (WHO) for zoonoses [5]. The programme aimed to achieve the prevention, detection, and control of TADs, addressing their regional and global dimensions through the combined strengths of the international organisations. SEACFMD and GF‐TADs facilitate regional alliances in TAD control, enabling capacity building and assisting in establishing programmes for the targeted control of specific TADs aligned with regional priorities [5]. FAO and OIE then developed a Global FMD Control Strategy, which was endorsed by representatives from more than 100 countries and by international and regional partners at the Second FAO/OIE Global Conference on FMD Control, held in Bangkok, Thailand, in 2012 [53].

The Global FMD Control Strategy, delivered through GF‐TADs and the SEACFMD campaign, provided a structured framework for endemic countries, including tools such as the Progressive Control Pathway for FMD (PCP‐FMD) [54] and WOAH’s Performance of Veterinary Services (PVS) evaluations. Together, these mechanisms supported incremental risk reduction, strengthened surveillance capacity, and alignment with international standards across the region.

The progress of the international FMD Global Control Strategy has been assessed at the regional level using roadmap platforms that enable the formulation of harmonised programmes. These enabled the exchange of information on virus circulation, diagnostic developments, vaccination, biosecurity, and other TAD control initiatives. Regional roadmaps were developed from coordinated actions within the seven recognised major FMD virus serotypes, with a long‐term shared control and eradication vision led by the GF‐TADs FMD working group, in collaboration with FAO and OIE regional offices, regional economic communities, regional organisations, EuFMD, and participating nations [5].

There has been substantial progress in SEAFMD, culminating in the SEACFMD campaign, which now includes China and, more recently, Mongolia [5]. Participants in the SEACFMD campaign have presented evidence of enhanced capacities of veterinary services, improved rural livelihoods, strengthened farm‐based economies, and increased transboundary trade in livestock and animal products across most GMS countries [5]. After completion of five phases of implementation, phase 6 of the SEACFMD campaign included an evaluation to provide guidance and inform future strategies for the ongoing campaign [5]. This included a review of the published roadmaps, PVS, evaluation documents, scientific literature, and other documents to assess progress systematically and a survey questionnaire to gather information on the performance of SEACFMD since its inception in 1997 [55]. The historical progress in Thailand, Lao PDR, and Cambodia is described as follows.

### 3.1. Thailand

Historical detail is included to illustrate how the long‐term, incremental development of diagnostics and surveillance shaped sustainable transboundary disease control capacity in an endemic setting. FMD has been recognised as a major constraint to livestock production in Thailand for several decades, and the country’s experience provides one of the most detailed historical examples of how the capacity for endemic transboundary disease control has evolved in SEA. Rather than rapid elimination, Thailand’s progress reflects incremental adaptation of diagnostics, surveillance, and vaccination policies in response to persistent virus circulation and cross‐border livestock movement [56, 57].

Formal efforts to strengthen FMD control in Thailand began in the mid to late 1980s through collaborative programmes involving the Department of Livestock Development (DLD) and international partners, notably the CSIRO Australian Animal Health Laboratory (AAHL), with support from the ACIAR [58, 59]. At that time, outbreak recognition relied largely on clinical diagnosis, with laboratory confirmation limited to complement‐fixation tests and strain characterisation dependent on external reference laboratories. Early programme objectives, therefore, focused on establishing in‐country diagnostic capability and improving understanding of the FMD virus strains circulating within Thailand.

A central component of this early phase was capacity building within DLD laboratories in Lampang and Pak Chong. The introduction of enzyme‐linked immunosorbent assay (ELISA)–based diagnostic platforms and serological assays during the late 1980s and early 1990s enabled more timely confirmation of outbreaks. This transition embedded laboratory diagnostics into national FMD surveillance systems and demonstrated that sustainable control in endemic settings depends on locally maintained and interpreted diagnostic tools [16, 17].

As diagnostic capacity improved, attention turned to characterising the diversity of FMD virus strains circulating in Thailand. Antigenic and molecular analyses conducted during the early 1990s revealed substantial variation among field isolates and frequent mismatches between circulating viruses and available vaccine strains, particularly for serotypes O [18], A [19], and Asia 1 [20]. Later studies evaluated the efficacy of newly introduced trivalent vaccines under field conditions, showing marked improvements in immune response following updated vaccination protocols [21]. These findings challenged the assumption that static vaccination programmes would be sufficient in a highly connected livestock system. They provided an early empirical basis for adapting vaccine strain selection in response to ongoing viral evolution and cross‐border introductions.

In parallel, field epidemiological studies were undertaken to better understand the drivers of FMD persistence at the village and production system levels. Studies conducted in northern and central Thailand identified consistent risk factors, including seasonal livestock movement, communal grazing, informal trade networks, and uneven vaccine coverage [22, 23]. These investigations highlighted that FMD persistence was shaped as much by livestock management practices and socioeconomic conditions as by virological factors, reinforcing the need to integrate epidemiological insights with laboratory data when designing control strategies.

Over time, the integration of diagnostic, epidemiological, and surveillance data enabled more effective translation of evidence into policy. Information on circulating serotypes and antigenic variation informed vaccination strategies, including the selection and deployment of vaccine strains and prioritisation of high‐risk areas. Thailand’s growing evidence base also supported engagement with regional initiatives, particularly through the SEACFMD campaign, reflecting recognition that national FMD control was unsustainable in isolation in a region characterised by frequent transboundary livestock movement [5]. It is noted that a PVS report on Thailand was published in 2012, identifying that there were fewer veterinarians in the field than adequate supporting staff structures at the national, provincial, or district level [5]. Control of FMD in Thailand has remained a priority, with the disease addressed through national programmes and the establishment of one FMD‐free zone. However, the PVS report identified deficiencies in vaccine production facilities and the constant threat of incursions to the newly established FMD‐free zone. In 2012, animal identification and traceability systems were still in early development [5].

The Thai FMD experience illustrates how long‐term adaptive development of diagnostics and surveillance can generate durable capacity for transboundary disease control in endemic settings. Rather than focused virus elimination, progress in disease control has been mainly achieved through sustained refinement of diagnostic tools, improved understanding of virus diversity and transmission, alignment of vaccination and surveillance strategies, increased funding commitment, and progressive adaptation to changes in regional livestock production systems and trade. These lessons remain directly relevant to other endemic settings facing similar technical, economic, and transboundary challenges. Despite these advances, important challenges remain. Informal livestock movement, heterogeneous vaccine coverage, incomplete animal traceability, and continued transboundary virus introductions continue to limit progress towards sustained FMD control. Consequently, improvements observed over time should be interpreted as incremental strengthening of disease management rather than evidence of elimination.

### 3.2. Lao PDR

Diagnostic development in Thailand provided the foundation for a more ambitious effort to understand FMD epidemiology in Lao PDR from the late 1990s onward. At that time, Laos lacked baseline data on FMD prevalence or serotype distribution, and the National Animal Health Laboratory (NAHL) was only beginning to expand its diagnostic capabilities. The ELISAs and QA systems validated in Thailand were implemented at NAHL and used to support multiple surveillance activities. A significant turning point came in 1999 when the PanAsia strain of serotype O swept through the Mekong subregion. Working collaboratively with WOAH, the International Livestock Research Institute (ILRI), and the national veterinary services, investigators undertook outbreak investigations, sample collection, and spread analysis [24], which informed the first structured serological surveys. These surveys provided statistically robust estimates of FMD seroprevalence. They confirmed widespread circulation of type O, with sporadic detections of types A and Asia 1 [25], guiding national vaccination strategies for years.

To address the challenges of field sampling in remote areas, an abattoir‐based surveillance model was developed in the late 1990s, enabling routine, low‐cost blood collection at slaughterhouses [26]. Nearly two decades later, this approach was successfully extended to FMD and expanded to other TADs [31, 32, 60]. This approach generated useful estimates of previous exposure while substantially reducing operational costs. However, abattoir populations may not represent source populations, vaccination history was usually unavailable, animal origins were sometimes uncertain, and serological data alone could not distinguish vaccination from previous infection or identify areas of active virus circulation. Consequently, abattoir surveillance was interpreted as complementary to outbreak investigation and virological surveillance rather than a replacement for either approach. Where possible, FMD serology was interpreted using nonstructural protein (NSP) testing and molecular investigations to improve epidemiological interpretation; however, complete traceback information and vaccine histories were rarely available under field conditions.

In parallel, collaboration with the World Reference Laboratory for FMD at Pirbright enabled the molecular characterisation of Lao outbreak samples. VP1 sequencing revealed two dominant type O topotypes: SEA and Middle East–South Asia (ME‐SA), alongside occasional incursions of types A and Asia 1 [27]. These findings confirmed the transboundary nature of FMD, driven by informal trade and livestock movement, and reinforced the need for regional vaccine banks, shared molecular surveillance, and harmonised control strategies.

Targeted investigations in northern Lao PDR identified persistent FMD ‘hotspots’ linked to animal movement, market networks, and transboundary trade, highlighting spatially clustered transmission dynamics and the limitations of passive surveillance alone [28]. Complementary vaccination trials suggested that strategic, risk‐based vaccination of large ruminants could significantly reduce clinical disease and transmission, providing supporting evidence that targeted immunisation is an effective and practical control strategy in resource‐limited, high‐risk settings [11].

These efforts established the first coherent epidemiological framework for FMD in Lao PDR, demonstrating how locally adapted diagnostics, abattoir‐based surveillance, outbreak investigation, and regional laboratory collaboration could underpin evidence‐based control strategies and inform coordinated transboundary disease management across the Mekong subregion. It is noted that Lao PDR requested PVS evaluation and gap analysis in 2011 and 2012. The gap analysis report recommended adopting interventions based on two strategies, especially for the PCP‐FMD [54] application. These included (i) an endemic disease‐oriented strategy aligned with the regional roadmap to prevent, control, and eradicate FMD and (ii) a market‐oriented strategy that can identify disease‐free zones for exporting cattle and buffaloes to the regional markets. The report suggested reducing the number of border crossings and equipping them with facilities for import/export inspections, with quarantine required. The gap analysis report suggested that Lao PDR should enter the PCP‐FMD and progress it and establish an animal identification and traceability system [5].

### 3.3. Cambodia

A 2011 longitudinal study in southern Cambodia reported that vaccination against FMD was rare, with less than 0.5% of large ruminants estimated to be protected, while outbreaks remained common during the study period [61]. FMD was frequently reported near the study project sites, especially in 2010, when disease reports increased significantly compared to 2008. However, no FMD cases were recorded during the study period in the villages that received FMD vaccination, despite the disease being common in nearby villages [61].

Economic‐ and livelihood‐focused research in Cambodia during the 2010s significantly advanced understanding of the impacts of FMD among smallholder cattle producers. Studies conducted in southern provinces quantified the substantial financial losses associated with FMD outbreaks, with affected households estimated to lose between USD $49 and $380 per event, often representing a major portion of annual income. These losses stemmed not only from treatment costs and reduced market value but also from indirect effects, such as a loss of draft power and delays in rice cultivation [29]. Broader analyses demonstrated that poorer households were disproportionately affected, highlighting the disease’s role in rural economic vulnerability and perpetuating disadvantage. Subsequent evaluations of best‐practice health and husbandry interventions found measurable improvements in livestock productivity and income where these interventions were adopted, supporting the integration of FMD control into wider rural development strategies [62]. Parallel studies examined the economic justification for national control strategies, revealing a strong return on investment in FMD vaccination and biosecurity education, with benefit‐cost ratios exceeding 3:1 in high‐risk areas [10]. Investigations into on‐farm biosecurity revealed low awareness of transmission risks and practical barriers to behaviour change among smallholder farmers, prompting the use of participatory and change management approaches to improve uptake. A focus on simple, locally feasible biosecurity measures, such as fencing, delayed animal movement, and improved isolation practices, was shown to reduce risk effectively when coupled with community‐based training and veterinary support [15, 30].

This body of work has contributed to a more sustainable, evidence‐driven approach to FMD control in Cambodia, linking economic impact assessment with behavioural science and policy‐relevant epidemiology. The study of a targeted extension and training programme for smallholder cattle farmers in southern Cambodia (2008–2010) showed significant improvements in farmers’ knowledge of cattle husbandry, disease recognition, biosecurity, and health measures following participatory training [61]. These gains improved animal health awareness and management, emphasising the importance of community‐based education on livestock health and biosecurity in low‐resource areas [61].

It is noted that a follow‐up PVS report on Cambodia, submitted in 2017, identified a range of relevant issues, including understaffing, limited resources, insufficient budgets for promoting new practices and implementing interventions, an inadequate supply of vaccines, and an overall weak veterinary service capability. Significant under‐reporting was identified, and laboratories required updated equipment to provide reliable test reports. Cambodia was among the countries in the region where it was suggested that serious efforts were needed to address the slow progress on the PCP‐FMD [5].

Building on the Lao experience, surveillance methodologies were subsequently extended into Cambodia, beginning with goats [63] and later expanding to cattle and pigs [33]. A series of studies provided the first national baseline for FMD seroprevalence across multiple livestock species, and abattoir‐based sampling proved effective when combined with field reports, offering a window into geographic risk mapping and vaccine planning [34]. Although these studies reported encouraging economic and behavioural outcomes, adoption of improved biosecurity and vaccination practices remained variable between communities and depended on sustained veterinary support, community engagement, and available resources. Long‐term maintenance of these gains requires continued investment beyond externally funded projects.

## 4. CSF

When efforts to investigate CSF began in Lao PDR in the late 1990s, the disease was poorly documented and not considered a national priority. Although suspected by field veterinarians, there were no reliable prevalence data, limited diagnostic capacity, and no structured evidence base. Initial investigations at the NAHL in Vientiane analysed 419 clinical submissions from suspected pig outbreaks between 1997 and 1999. CSF virus (CSFV) was detected in 25.1% of samples, confirming circulation in 12 of Laos’s 18 provinces [35]. The burden was highest in central and southern regions, where pig densities and informal animal movements were higher. For the first time, CSF distribution could be mapped with confidence, transforming it from a suspected problem into a clearly documented, nationwide disease. A summary of studies is presented in Table 1.

### 4.1. Molecular Characterisation

Molecular characterisation of CSFV has provided key insights into viral diversity and cross‐border transmission dynamics in mainland SEA. In Lao PDR, sequencing of the E2 glycoprotein gene and the 5′ noncoding region (NCR) from outbreaks in the late 1990s and early 2000s identified two cocirculating CSFV subgenotypes: subgenotype 2.1 predominantly in northern and central areas and subgenotype 2.2 in the south [37, 38]. This observation reflects connections with neighbouring Vietnam, Thailand, and southern China and points to cross‐border pig movements as a driver of transmission, as well as to limited inter‐regional pig movements within Laos itself.

In Thailand, CSFV genotyping has historically documented the circulation of multiple viral lineages, with genotype 2, particularly subgenotype 2.2, becoming the dominant circulating group from the mid‐1990s onwards, replacing earlier genotypes 1.1 and 1.3 [43, 44]. Although comprehensive, contemporary national sequence datasets remain limited in the public domain, this pattern is consistent with regional CSFV ecology and phylogenetic studies that place Thai isolates within a broader genotype 2 landscape shared across mainland SEA and southern China [46–48]. In Vietnam, sustained circulation of subgenotypes 2.1 and 2.2 has been documented, with frequent lineage turnover and evidence of repeated introductions from adjacent provinces in China [45]. In southern China, genotype 2, particularly 2.1 variants, has become dominant over the past two decades, replacing earlier genotype 1 lineages and seeding outbreaks across borders through formal and informal pig movements [46–48].

Together, these data confirm that genotype 2 remains the predominant viral group in both Lao PDR and Thailand, with subgenotypes 2.1 and 2.2 cocirculating in Lao PDR [37, 38] and subgenotype 2.2 historically dominant in Thailand [44]. Phylogenetic placement of Lao and Thai isolates within Vietnamese and southern Chinese clades underscores that CSF in mainland SEA is shaped by cross‐border livestock movement and shared epidemiological pressures. This regional structure means that viral evolution in southern China and northern Vietnam is likely to be rapidly reflected in Lao and Thai pig populations, underscoring that CSF cannot be effectively managed within national boundaries alone.

### 4.2. Population‐Level Surveillance

These early diagnostic and molecular findings laid the foundation for broader discussions on harmonised surveillance and control in Lao PDR. To understand the broader epidemiological picture, population‐level serological surveys were conducted across different agroecological zones. These surveys, while limited in scope and resource‐intensive, aimed to assess both viral exposure and the coverage of existing vaccination programmes. The results revealed a heterogeneous landscape: Peri‐urban areas showed moderate levels of protective antibodies, indicating partial vaccine uptake, whereas remote rural districts displayed very low antibody prevalence, reflecting limited access to vaccination and minimal awareness of CSF among farmers [35]. The detection of antibodies in unvaccinated pigs further suggested ongoing viral circulation and the likelihood of subclinical and endemic transmission.

The surveys highlighted three major gaps: inconsistent vaccine coverage, uncertainty regarding vaccine quality or efficacy, and uneven veterinary extension services, particularly in rural areas. Additionally, systems for monitoring vaccine uptake and effectiveness were limited. These insights informed recommendations to improve vaccine access and cold‐chain management and to strengthen community engagement to support CSF prevention and control [35, 36].

Given the logistical and financial challenges of large‐scale field sampling, an abattoir‐based surveillance approach was introduced. In 1999, a pilot study across six provinces used slaughterhouses as sentinel sites for serological testing. This method proved efficient, cost‐effective, and readily adopted by local veterinary staff. Seroprevalence ranged from 12% to 29% [35], confirming active CSF circulation across multiple regions. Notably, the model proved sustainable: When repeated in 2019 as part of a longitudinal study [42], seropositivity had increased to 68.7%, possibly reflecting improved vaccine coverage, although vaccination records were unavailable, and other explanations, including changing slaughter populations and natural infection, cannot be excluded. However, this has not been conclusively proven due to a lack of vaccination data. Comparing results over a 20‐year period provided rare insights into the trajectory of CSF control and demonstrated the value of abattoir‐based surveillance as a long‐term monitoring platform.

### 4.3. Diagnostic Adaptation for Low‐Resource Settings

Another critical area of work involved strengthening diagnostics. Laboratory capacity in Lao PDR was still developing, and many protocols relied on reagents, equipment, and cold‐chain conditions that were difficult to maintain. To address these constraints, several studies evaluated the performance of reverse transcription‐polymerase chain reaction (RT‐PCR) and antigen‐capture ELISA under local conditions, examining issues such as RNA degradation, sample handling, reagent stability, and workflow feasibility [39, 40, 64]. These assessments provided practical guidance for laboratories attempting to detect CSF reliably despite infrastructural limitations. One further innovation was the development and testing of an immunomagnetic bead assay for CSF antigen detection, which required minimal equipment, produced rapid results, and proved suitable for low‐resource laboratories [41].

### 4.4. Scaling Surveillance Across Borders

Building on lessons from Lao PDR, an abattoir‐based active surveillance model was piloted in Cambodia, generating the country’s first structured seroepidemiological data on CSF. Despite limited resources, Cambodian veterinary services successfully adapted the Lao methodology, demonstrating its regional applicability. The programme also integrated data collection for CSF, PRRS, and FMD, providing a proof‐of‐concept for multidisease surveillance platforms that can function within constrained veterinary systems [33].

In summary, the CSF work in Lao PDR and Cambodia shows how a stepwise, pragmatic approach, from basic diagnostic confirmation to molecular epidemiology and multidisease surveillance, can transform national understanding of an endemic disease. It underscores the value of appropriate technology and partnership‐based research in building scalable, sustainable systems and highlights that tools tailored to local needs are far more likely to be adopted and institutionalised over time.

## 5. ASF

In mid‐2019, the detection of ASF in northern Lao PDR marked a pivotal moment in the region’s experience with TADs. Until then, ASF had been a looming threat advancing through China and Vietnam. Once confirmed in Laos, the response shifted abruptly from preparedness to crisis management. Unlike FMD or CSF, ASF currently has no widely applicable field vaccination strategy, and control depends primarily on early detection, movement control, biosecurity, and rapid response. The environmental persistence of the ASF virus and the movement of contaminated pork products further complicate control. Mortality was extremely high, and the consequences for rural livelihoods, food security, and the national economy were immediate and severe. The ASF incursion tested whether decades of diagnostic and surveillance investment could be repurposed for an unprecedented emergency. A summary of studies is presented in Table 1.

### 5.1. Diagnostics for Resource‐Limited Contexts

The immediate priority was improving access to diagnostics. Confirming ASF required PCR testing at central laboratories, but long transport routes, unreliable cold chains, and administrative delays often meant results arrived days later, far too slow for a disease capable of wiping out herds within 48–72 h. To close this gap, a commercially available, low‐cost rapid diagnostic test (RDT) for ASF antigen was evaluated under rural Lao conditions [49]. This was the first field validation of its kind in the country and among the earliest in the region. The RDT showed high specificity and acceptable sensitivity in real‐world settings. Although not a substitute for PCR, it proved valuable as a triage tool, enabling veterinarians in remote districts to quickly identify suspect cases, advise farmers, and trigger local response actions. Notably, the test required no electricity, cold chain, or specialised equipment, making it well‐suited to the areas where ASF was most likely to emerge and significantly extending the diagnostic reach of the veterinary system.

### 5.2. Epidemiology and Livelihood Impact

The impact of ASF in Laos quickly proved to be far more than a veterinary issue. As outbreaks moved through rural districts, it became evident that ASF impact was a major socioeconomic shock, particularly for smallholder and ethnic minority communities that relied on pigs as savings, income, and social capital. To capture these broader effects, a mixed‐methods study assessed the socioeconomic consequences of ASF outbreaks [50]. Interviews, surveys, and community workshops revealed cascading disruptions following herd loss: reduced income, rising household debt, cancelled education expenses, and, in some cases, outmigration. Female‐headed households and ethnic minority groups were disproportionately affected due to their dependence on pig production and limited access to veterinary support. These findings reframed ASF as a multidimensional development crisis, underscoring the need for integrated approaches that incorporate animal health into social and economic resilience.

### 5.3. Surveillance Innovations

Building on the success of abattoir‐based surveillance for FMD and CSF, the collaborating investigators assessed whether a similar model could support ASF monitoring [42]. Although the high mortality of ASF meant few infected pigs reached slaughter during active outbreaks, the postepidemic period offered an opportunity to detect residual virus circulation. Working with provincial veterinary services, abattoir‐based sampling was implemented in several provinces, with testing for ASF antibodies and antigens providing a low‐cost means of assessing ongoing transmission in previously affected areas. The approach generated valuable data to define zones of disease freedom, inform quarantine releases, and support the safe resumption of trade. By integrating ASF monitoring into a broader multidisease platform, including CSF, PRRS, and other swine pathogens, veterinary authorities with limited budgets improved surveillance efficiency and gained a more comprehensive picture of swine health. Despite these advances, substantial uncertainty remains regarding ASF epidemiology in smallholder systems. Under‐reporting, informal slaughter, fear of uncompensated culling, illegal pig movement, and limited passive surveillance sensitivity continue to obscure the true burden and transmission dynamics of ASF throughout the region.

### 5.4. Transmission Dynamics

One of the most technically challenging components of the ASF response was reconstructing the epidemic’s transmission dynamics. Using detailed outbreak data from Oudomxay Province, one of the earliest and hardest‐hit areas, the collaborating investigators developed a spatiotemporal model of ASF spread [51]. Incorporating household pig ownership, biosecurity practices, geographic proximity, and outbreak timing, the model revealed a fast‐moving transmission pattern driven by common practices such as free‐ranging pigs, swill feeding, and communal slaughter. The estimated basic reproduction number (*R*_0_) was high, indicating that ASF could spread rapidly in unregulated smallholder systems. The analysis also highlighted substantial delays between the onset of clinical signs and official reporting, often linked to logistical barriers, fear of uncompensated culling, and limited disease recognition. These delays allowed undetected viral spread and significantly hindered containment efforts.

### 5.5. Policy Translation and Preparedness

Finally, the project culminated in translating data into policy tools. Surveillance results and transmission models were developed in collaboration with partners in Lao PDR, where they were used by the Department of Livestock and Fisheries (DLF) to inform outbreak response protocols and national preparedness plans [52]. A scenario‐based model informed by evidence from formal disease reporting mechanisms, including WOAH’s World Animal Health Information System (WAHIS), helped guide the strategic placement of quarantine checkpoints, resource allocation for diagnostic support, and risk communication campaigns. By embedding this technical work into the policy process, the collaborating investigators helped shift the ASF narrative from reactive emergency response to proactive risk management. These outputs were incorporated into national preparedness planning by the DLF and informed decisions on surveillance priorities, quarantine placement, and diagnostic resource allocation.

### 5.6. Cross‐Cutting Innovations and Lessons Learned

These observations represent a synthesis of heterogeneous studies and programme experience and should be interpreted as context‐dependent lessons rather than causal conclusions. Controlling TADs in SEA requires more than individual diagnostic tools or discrete surveillance projects. Across Thailand, Lao PDR, and Cambodia, the reviewed studies suggest that gains were more likely to be sustained when technical solutions aligned with local surveillance and response capacities, collaborative regional epidemiology, and shared advocacy and policy realities [5]. Several cross‐cutting lessons were consistent across the four decades of work described.

### 5.7. Diagnostics That Fit the System

The reviewed studies suggest that diagnostic tools were particularly useful when designed around local constraints rather than ideal conditions. Replacing virus‐neutralisation tests with serotype‐specific ELISAs for FMD, coupled with the introduction of quality control methodologies, demonstrated that simple, scalable tools could enhance confidence in laboratory results even in settings with limited infrastructure. Similar principles guided later work in Lao PDR, where affordable RT‐PCR protocols and antigen detection assays were stress‐tested under real‐world conditions and where RDTs for ASF enabled rapid triage in remote districts. Across the studies reviewed, these findings suggest that diagnostics should be scientifically robust, operationally feasible, and maintainable without continued external support. Nevertheless, long‐term sustainability depends upon maintaining laboratory infrastructure, reagent supply chains, and trained personnel after donor‐funded programmes conclude.

### 5.8. Surveillance as a Scalable Platform

Abattoir‐based serosurveillance proved to be one of the most efficient and sustainable platforms for routine monitoring of TADs. First piloted for FMD and then expanded to CSF, ASF, Q fever, brucellosis, and melioidosis, the approach generated national‐scale datasets, although representativeness was limited by slaughter‐population selection at far lower operational cost than widespread field sampling. The ability to compare CSF seroprevalence across a 20‐year timespan highlights its value as a long‐term monitoring system. Importantly, abattoir‐based surveillance complemented rather than replaced field investigations and passive reporting systems, enabling countries to build layered, evidence‐driven surveillance architectures within existing budgets. However, abattoir‐based surveillance has the significant disadvantage of lacking ancillary data, particularly the vaccination status for TADs. Additional limitations include sampling bias towards animals entering formal slaughter systems, incomplete traceability of animal origin, underrepresentation of remote production systems, and inability to estimate disease incidence directly. These limitations reinforce the importance of integrating abattoir surveillance with field investigations, passive surveillance, and molecular epidemiology.

### 5.9. Molecular Epidemiology and Regional Harmonisation

Genetic characterisation consistently revealed that pathogen movement reflected poor biosecurity at international boundaries. FMD viruses in Laos showed clear links to strains circulating in Vietnam and China, with CSF subgenotypes reflecting broader regional trade and animal movement patterns. These findings emphasise the need for harmonised regional vaccine strategies, shared molecular surveillance data, and coordinated outbreak responses. Molecular epidemiology has provided both technical insights and a shared language through which countries could align control measures.

### 5.10. Workforce Development and System Resilience

The programme experience indicates that human capacity was an important factor in the sustainability of diagnostic, surveillance, and response systems. Training‐of‐trainers programmes, biosafety and biosecurity courses, the introduction of biorepository management, provincial laboratory assessments, necropsy training, applied livestock health and production field studies for veterinarians and community animal health workers, field‐level biosecurity and vaccination initiatives, development and distribution of livestock husbandry extension materials, and introduction of modern livestock management techniques have collectively strengthened institutional capability. These activities supported national and provincial laboratories and field services in adopting new diagnostics, maintaining quality assurance systems, conducting surveillance investigations, and responding to TAD outbreaks. Investments in personnel development appear to have been important for sustaining these gains [5]. However, retention of trained personnel remains difficult in several countries because of resource limitations, staff turnover, and dependence on externally funded programmes.

### 5.11. Turning Data Into Decisions

The examples reviewed suggest that collaboratively generated, timely, and transparently shared data may be more likely to influence policy. Policy relevance appeared greatest when national authorities were directly involved in interpreting and reporting the results. Effective vaccination depends not only on antigenic matching between vaccine strains and circulating viruses but also on vaccine potency, batch consistency, quality assurance, cold‐chain integrity, delivery, coverage, and postvaccination evaluation [5, 21]. CSF seroprevalence maps guide vaccination priorities; however, incomplete or absent vaccination records make it difficult to determine whether low antibody prevalence reflects inadequate coverage, vaccine quality, cold‐chain or administration problems, or variation in immune response [35, 36]. This collaborative approach strengthens ownership, facilitates advocacy and policy uptake, and integrates scientific outputs into national and international decision‐making processes.

### 5.12. Integration With Regional and International Frameworks Amplifies Impact

Partnerships with FAO, WOAH, the U.S. DTRA, ACIAR, and national institutes were critical in harmonising laboratory methods, sharing reagents, enabling molecular surveillance, facilitating epidemiological findings to inform vaccination and biosecurity strategies, and embedding national findings into regional disease control campaigns. These collaborations, particularly the PVS and PCP initiatives delivered through WOAH, supported cross‐cutting capacity building while ensuring that national systems aligned with international standards, enhancing preparedness for both endemic and emerging diseases. Implementation has nevertheless remained heterogeneous across countries owing to differences in veterinary capacity, funding, governance, and competing national priorities.

### 5.13. Sustaining Progress Requires Stable Financing and Political Commitment

Much progress in abattoir surveillance, diagnostics, biosafety, epidemiology, biosecurity, and vaccination was driven by long‐term external investment. The SEACFMD evaluation highlighted 15 priority areas, including disease surveillance, biosecurity, animal movement, border quarantine, FMD vaccination, public awareness, emergency response, political and financial commitments, and regional policy. It also emphasised improving veterinary services and public trust issues [5]. Recent funding cuts threaten ongoing efforts, especially in low‐resource regions with fragile surveillance and response. While long‐term external investment has substantially strengthened surveillance and laboratory capacity, donor dependence remains an important vulnerability. Future sustainability will require greater national investment, stronger public–private partnerships, and continued regional collaboration. This highlights the need for societal and political advocacy for a regional livestock food security system that promotes national ownership, regional coordination, and external support to empower smallholder farmers. Greater engagement of commercial producers, livestock traders, producer organisations, and vaccine manufacturers will also be important for sustaining disease surveillance, improving biosecurity, and supporting long‐term regional control programmes.

## 6. Limitations, Remaining Challenges, and Future Priorities

This review provides a narrative synthesis of four decades of TAD research and capacity development undertaken primarily in Thailand, Lao PDR, and Cambodia. Although it draws extensively on peer‐reviewed publications, it also incorporates programme reports, institutional knowledge, and the experiences of authors who participated directly in many of the activities described. Consequently, the review reflects both the strengths and limitations of a narrative approach. Unlike a systematic review, studies were not selected using predefined inclusion criteria or formal quality assessment, and emphasis was placed on research that contributed to the progressive development of diagnostics, surveillance, and disease control within the GMS. While every effort was made to provide a balanced interpretation, some degree of selection or interpretive bias is inherent in narrative reviews and cannot be completely excluded. Furthermore, many important regional initiatives undertaken by national veterinary services, FAO, WOAH, the European Union, Japan, China, the Republic of Korea, and other international partners are beyond the scope of this review.

Several limitations also apply to the surveillance systems described. Abattoir‐based surveillance has proven to be a practical, cost‐effective, and sustainable approach for monitoring multiple endemic diseases in resource‐limited settings, particularly where active field surveillance is difficult to maintain. However, slaughter populations may not be fully representative of the wider livestock population, and important epidemiological information, including vaccination history, animal origin, management practices, and movement history, is frequently unavailable. Serological data alone cannot distinguish recent infection from historical exposure or vaccination without complementary laboratory testing, and these systems are generally better suited to estimating patterns of exposure than to identifying active transmission. Consequently, abattoir‐based surveillance should be regarded as one component of an integrated surveillance framework that also includes outbreak investigation, molecular epidemiology, passive surveillance, and targeted field studies.

Despite substantial advances in diagnostics, epidemiology, and laboratory capacity, important uncertainties remain regarding the epidemiology and control of major TADs in SEA. Informal cross‐border livestock movement, incomplete animal identification and traceability systems, heterogeneous vaccination coverage, and variable veterinary service capacity continue to facilitate pathogen spread throughout the region. For ASF in particular, under‐reporting, informal slaughter, movement of contaminated pork products, limited compensation mechanisms, and delays in disease reporting continue to hinder accurate assessment of disease burden and transmission dynamics. Similarly, interpretation of serological data for diseases such as FMD and CSF remains challenging where vaccination histories are incomplete and where vaccine strain matching, potency, quality assurance, cold‐chain integrity, coverage, and administration vary between locations and over time. Limited batch‐level information and postvaccination monitoring frequently make it difficult to determine whether inadequate population immunity reflects vaccine quality, programme delivery, incomplete coverage, or natural variation in immune response.

Long‐term sustainability also remains a significant challenge. Much of the progress described in this review has been supported through sustained investment by national governments and international development partners. These investments have strengthened laboratory infrastructure, surveillance systems, workforce development, and regional collaboration over several decades. However, continued dependence on external funding creates vulnerabilities, particularly where national investment remains constrained. Maintaining diagnostic capability, quality assurance systems, surveillance networks, and trained personnel requires long‐term political commitment and stable financial support beyond individual project cycles. Greater engagement with the private livestock sector, producer organisations, and commercial value chains will also be important for improving biosecurity, disease reporting, and implementation of risk‐based disease control measures.

Future progress will depend increasingly on strengthening regional integration rather than relying solely on national disease control programmes. Continued harmonisation of surveillance systems, molecular epidemiology, laboratory quality assurance, harmonised vaccine‐quality standards, and independent quality control procedures, including appropriate potency and batch‐release testing, cold‐chain monitoring, postvaccination evaluation and regional sharing of vaccine‐performance data, animal identification and traceability systems, and cross‐border data sharing, will be essential for addressing diseases that routinely transcend national boundaries. Advances in molecular diagnostics, genomic surveillance, digital epidemiology, and geospatial risk analysis offer important opportunities to improve outbreak detection and support evidence‐based decision‐making. However, the greatest gains are likely to arise from integrating these technologies with strong veterinary services, effective community engagement, and locally appropriate disease management strategies. Ultimately, sustainable control of TADs in the GMS will depend not only on scientific innovation but also on continued regional cooperation, resilient veterinary institutions, and long‐term investment in animal health systems.

## 7. Conclusion

Over four decades, the integration of diagnostics, surveillance, epidemiology, and response systems has strengthened regional capacity to manage TADs. Nevertheless, capacity remains uneven, and informal livestock movement, variable vaccination coverage and quality, incomplete traceability, under‐reporting, workforce constraints, and dependence on external funding remain important limitations. Parts of SEA may therefore now be better equipped to detect and manage some disease threats, provided that surveillance, laboratory capability, vaccine‐quality systems, and regional coordination are sustained.

## Author Contributions

Stuart D. Blacksell conceived the review and led the overall conceptual development, synthesis, and writing of the manuscript. Peter A. Windsor and Michael P. Ward contributed to the conceptual framing, interpretation of historical and regional transboundary animal disease control programmes, and critical revision of the manuscript. James R. Young, Russell D. Bush, Harish Tiwari, Nina Matsumoto, James V. Conlan, and Michael P. Ward contributed to the interpretation of epidemiological, socioeconomic, and surveillance data and critically revised the manuscript. Syseng Khounsy, Phouvong Phommachanh, Sothyra Tum, Somjai Kamolsiripichaiporn, Jaison B. Kolenchery, Jarunee Siengsanan‐Lamont, and Laurence J. Gleeson contributed to laboratory, surveillance, field epidemiology, and diagnostic programme development described in the review and assisted with data interpretation and manuscript revision.

## Funding

This research was funded in part by the Wellcome Trust (Grant 315982/Z/24/Z). For the purpose of open access, the author has applied a CC BY public copyright license to any author accepted manuscript version arising from this submission.

## Disclosure

The authors reviewed, edited, and verified all generated content and accept full responsibility for the content of this publication. All authors reviewed and approved the final manuscript. This article is based on previously published studies, surveillance reports, and programme data cited within the manuscript.

## Conflicts of Interest

The authors declare no conflicts of interest.

## Data Availability

No new datasets were generated or analysed during the current study.
